# Enhanced secretion of hepatocyte growth factor in human umbilical cord mesenchymal stem cells ameliorates pulmonary fibrosis induced by bleomycin in rats

**DOI:** 10.3389/fphar.2022.1070736

**Published:** 2023-01-06

**Authors:** Huanjie Chen, Yulong Luo, Yiping Zhu, Yongshun Ye, Difei Chen, Xinyu Song, Zhulin Xiao, Ming Liu, Shiyue Li

**Affiliations:** ^1^ State Key Laboratory of Respiratory Disease, National Clinical Research Center for Respiratory Disease, Guangzhou Institute of Respiratory Health, The First Affiliated Hospital of Guangzhou Medical University, Guangzhou, Guangdong, China; ^2^ Innovation Centre for Advanced Interdisciplinary Medicine, Key Laboratory of Biological Targeting Diagnosis, Therapy and Rehabilitation of Guangdong Higher Education Institutes, The Fifth Affiliated Hospital of Guangzhou Medical University, Guangzhou, Guangdong, China; ^3^ Huizhou Municipal Central Hospital, Guangzhou, Guangdong, China

**Keywords:** umbilical cord, mesenchymal stem cells, pulmonary fibrosis, hepatocyte growth factor, interleukin-17

## Abstract

Umbilical cord mesenchymal stem cells (UCMSCs) are a reportedly promising choice in the treatment of irreversible pulmonary fibrosis and lethal interstitial lung disease with limited drug treatment options. In this study, we investigated the therapeutic efficacy of UCMSCs overexpressing hepatocyte growth factor (HGF), which is considered one of the main anti-fibrotic factors secreted by MSCs. Adenovirus vector carrying the HGF gene was transfected into UCMSCs to produce HGF-modified UCMSCs (HGF-UCMSCs). Transfection promoted the proliferation of UCMSCs and did not change the morphology, and differentiation ability, or biomarkers. Rats were injected with HGF-UCMSCs on days 7 and 11 after intratracheal administration of bleomycin (10 mg/kg). We performed an analysis of histopathology and lung function to evaluate the anti-fibrotic effect. The results showed that HGF-UCMSCs decreased the Ashcroft scores in hematoxylin and eosin-stained sections, the percentage positive area in Masson trichrome-stained sections, and the hydroxyproline level in lungs. Forced expiratory volume in the first 300 m/forced vital capacity was also improved by HGF-UCMSCs. To explore the possible therapeutic mechanism of HGF-UCMSCs, we detected inflammatory factors in the lungs and performed mRNA sequencing in UCMSCs and HGF-UCMSCs. The data indicated that inhibition of interleukin-17 in the lung may be related to the anti-fibrosis of HGF-UCMSCs, and overexpressed HGF probably played a primary role in the treatment. Collectively, our study findings suggested that the overexpression of HGF may improve the anti-fibrotic effect of UCMSCs through directly or indirectly interacting with interleukin-17-producing cells in fibrotic lungs.

## Introduction

Idiopathic pulmonary fibrosis (IPF) is one of the forms of chronic progressive fibrotic interstitial lung disease (Lederer and Martinez, 2018). Patients with IPF eventually develop and ultimately die of irreversible respiratory failure (Richeldi et al., 2017). The death rate among patients with IPF has gradually increased, with an annually increasing trend (Hutchinson et al., 2014; Raghu et al., 2014; Hutchinson et al., 2015; Dove et al., 2019; Navaratnam and Hubbard, 2019; Tran et al., 2020). At present, the most effective treatment is lung transplantation, but transplant rejection and a lack of donor’s lungs make it impossible for more patients to benefit from transplantation (Raghu et al., 2015; George et al., 2019). Although pirfenidone and nintedanib can improve lung function and exercise tolerance to some extent, it is still difficult to prevent IPF from progressing (King Jr et al., 2014; Richeldi et al., 2014). Thus, new curative methods for patients with IPF are urgently needed.

Mesenchymal stem cells (MSCs) have been widely proven to effectively treat pulmonary fibrosis in animals through secreting anti-fibrotic factors (Huang et al., 2015; Kotani et al., 2017; Chen et al., 2018). Given that MSCs were likely an effective way to cure patients with IPF, clinical studies were subsequently conducted. The results showed that MSC administration was safe and could delay the deterioration of pulmonary function over time (Tzouvelekis et al., 2013; Chambers et al., 2014; Glassberg et al., 2017). Nevertheless, the progression of pulmonary fibrosis still cannot be stopped. Therefore, researchers must find ways of enhancing the anti-fibrotic efficacy of MSCs.

Gene modification of anti-fibrotic factors is a valid means to achieve the abovementioned goal (Madrigal et al., 2014; Ocansey et al., 2020). The hepatocyte growth factor (HGF) is a crucial factor in anti-fibrosis (Dong et al., 2015; Cahill et al., 2016). It has been shown that HGF can repair damaged alveolar epithelium and inhibit the profibrotic ability of fibroblasts and myofibroblasts in the lungs (Mizuno et al., 2005; Lee et al., 2008; Shukla et al., 2009). Umbilical cord MSCs (UCMSCs) are most likely the appropriate choice for modification due to their naturally high secretion of HGF, abundant supply, and no invasive extraction or donor site morbidity (Prasanna et al., 2010; Balasubramanian et al., 2012; Han et al., 2013; El Omar et al., 2014; Marmotti et al., 2017; Kim et al., 2018; Al Naem et al., 2020). HGF-modified UCMSCs (HGF-UCMSCs) have been used in the treatment of bronchiolitis obliterans, wound healing, acute kidney injury, and liver fibrosis, among others (Chen et al., 2011; Li et al., 2015; Cao et al., 2016; Yin et al., 2020). However, the application of HGF-UCMSCs in the treatment of bleomycin-induced pulmonary fibrosis has rarely been reported. A comparison of the therapeutic effects between HGF-UCMSCs and identified anti-fibrotic drugs is also needful.

In the present study, we evaluated the therapeutic outcome of HGF-UCMSCs in rats with pulmonary fibrosis induced by bleomycin. The therapeutic mechanism of HGF-UCMSCs was also explored using lung cytokines detection together with mRNA sequencing.

## Materials and methods

### Isolation and culture of human UCMSCs

Clinical-grade UCMSCs were obtained from Beijing SH Biotechnology (http://www.bjshbio.com/). The isolation and culture of UCMSCs were based on previously described methods (Chu et al., 2019), as follows. The umbilical cord was minced into small pieces then washed thoroughly with phosphate buffer saline (PBS) (Gibco, United States) and digested using collagenase (Gibco, United States) at 37°C for 60 min. The digestion was stopped using an MSCs growth medium (Beijing SH Technology, China), and the digested mixture was passed through a 70-μm cell strainer (BD, United States) to obtain a single-cell suspension. All primary UCMSCs were seeded in flasks at a density of 8,000/cm^2^ and cultured at 37°C in a humidified atmosphere containing 5% CO2.

### Production of HGF-UCMSCs

The protocol for producing HGF-UCMSCs was referred to in the previous paper (Wang et al., 2013). A replication-defective adenovirus expressing human HGF (Ad-HGF) and a replication-defective adenovirus not carrying exogenous genes (Ad-Null) were constructed with the AdEasy system (Stratagene, United States) and were purified by double cesium chloride density gradient ultracentrifugation. Ad-HGF and Ad-Null dissolved in storage buffer (Hanks’ buffer, 10% glycerol) were stored at −80°C. According to the previous protocol, UCMSCs were infected with 150 multiplicities of infection of Ad-Null or Ad-HGF. The cells were collected 48 h post-infection for further usage in vitro and *in vivo* experiments. Before treatment, the conditioned medium of MSCs infected with Ad-Null and Ad-HGF was collected for HGF testing according to the instructions in an HGF ELISA kit (ExCell, China). Medium not used for cell culture was utilized as a negative control in the ELISA reaction.

### Identification of MSCs

Cultured MSCs were identified for cell morphology and adherence, immune surface markers, and differentiation potential (Nadri et al., 2002; Soleimani and Nadri, 2009). MSCs were photographed for observation using a fluorescence digital microscope BZ-X800 (Keyence, Japan). Immune surface markers (i.e., CD105, CD73, CD90, CD34, CD11b, CD19, CD45, and HLA-DR) were analyzed by flow cytometry using a Human MSC Analysis Kit (BD, United States). For osteogenesis or adipogenesis, MSCs were respectively incubated in an osteogenic or adipogenic medium (Cyagen, China) for 3 weeks, and were then fixed with methanol. A Leica DMI 3000B fluorescence microscope (Leica, Germany) was used for photographs of osteoblasts or adipoblasts stained with alizarin red or Oil Red O, respectively.

### Animals and experimental design

Sprague-Dawley rats (6 weeks, 200–220 g) were purchased from Guangdong Medical Laboratory Animal Center (China) and were housed in a specific pathogen-free animal facility. All studies were approved by the Animal Ethics Committee of Guangdong Medical Laboratory Animal Center.

**FIGURE 1:**
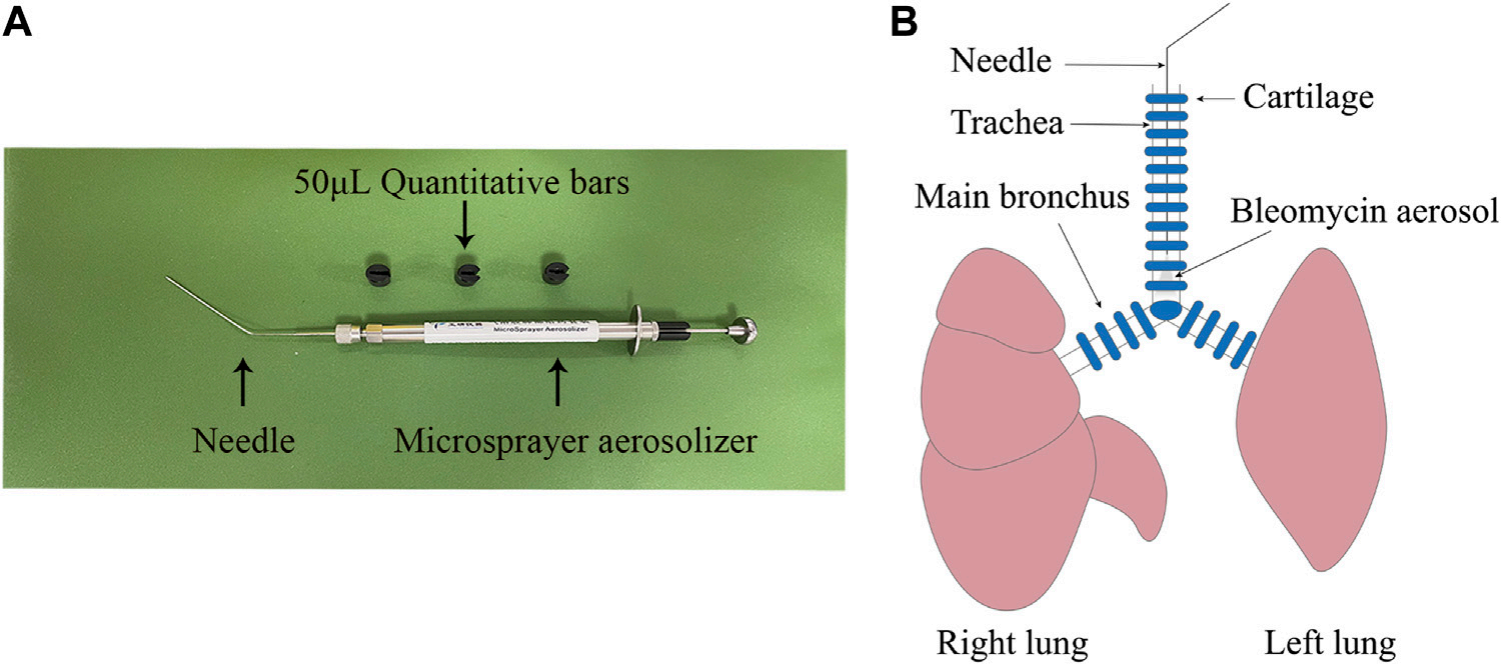
Establishment of pulmonary fibrosis model. **(A)** A microsprayer aerosolizer was used to deliver aerosols of PBS or bleomycin into the lungs of rats **(B)** The aerosols were presented in the lower tracheas.

Rats were divided into five groups (six rats per group): a CTRL group, BLM group, UCMSC group, HGF-UCMSC group, and PFD group. A microsprayer aerosolizer (Yuyan Instruments Co., Ltd. China) (Figure 1) was used to deliver sterile PBS or bleomycin into the lungs. On day 0, rats in the CTRL group were instilled intratracheally with sterile PBS; the remaining rats were instilled with 10 mg/kg body weight bleomycin (Hanhui co. LTD., China). From day 7–20, rats in the PFD group were infused orally with 100 mg/kg body weight pirfenidone once a day. The remaining rats were injected with sterile PBS, UCMSCs, or HGF-UCMSCs *via* tail vein on both days 7 and 11, as follows. 1) CTRL group and BLM group: sterile PBS, 400 μL/day per rat; 2) UCMSC group: UCMSCs, 2 * 10^6/400 μL/day per rat; 3) HGF-UCMSC group: HGF-UCMSCs, 2 * 10^6/400 μL/day per rat.

For the collected lungs on day 21, left lungs were inflated and immersed with 4% paraformaldehyde (Biosharp, China), and right lungs were frozen at −80°C in a refrigerator as quickly as possible.

### Lung function test

Rats were anesthetized with pentobarbital sodium and endotracheal intubation was performed. The intubated catheter was connected to a Buxco pulmonary function testing system (DSI Buxco, United States) to measure lung function as follows: forced expiratory volume in the first 300 m/forced vital capacity (FEV300/FVC), peak expiratory flow (PEF), chord compliance (Cchord), and total lung capacity.

### Hydroxyproline evaluation

The right lung lobes of each rat were ground into a powder in liquid nitrogen and used for hydroxyproline (HYP) detection. To assess the total collagen content of lung tissue, we used an HYP assay kit (Nanjing Jiancheng Bioengineering Institute, China). The experiment was performed according to the manufacturer’s instructions.

### Cytokine detection

Some lung tissue powder was prepared for cytokine detection, referring to the protocol of the Rat Cytokine/Chemokine Magnetic Bead Panel (Millipore, United States). Interleukin-17 (IL-17), IL-10, vascular endothelial growth factor (VEGF), and granulocyte-macrophage colony-stimulating factor (GM-CSF) were detected in 96-well plates using MILLIPLEX^®^ MAGPIX with MILLIPLEX Analyst. V5.1 software. Median fluorescence intensity data were analyzed using a five-parameter logistic method for calculating analyte concentrations in samples.

### Histopathological analysis

Left lungs were processed by Bios Biological Co. Ltd. (China). For paraffin sections, lungs were dehydrated using gradient ethanol and then embedded in paraffin blocks. The blocks were cut into sections of 3–5 μm thickness; sections were placed on polylysine-coated glass slides and stored at room temperature for further use. Hematoxylin and eosin (H&E) and Masson trichrome staining were performed following the standard protocol. The stained sections were captured, and the pictures were sent to two pathologists for evaluation. The severity of the injury was quantified using the Ashcroft scoring system in H&E-stained lung sections (Ashcroft et al., 1988). The percentage of blue-stained area in Masson trichrome-stained lung sections was quantified using ImageJ (National Institutes of Health, United States).

### mRNA sequencing

UCMSCs (*N* = 3) and HGF-UCMSCs (*N* = 3) were lysed and total RNA was extracted using a Trizol reagent kit (Invitrogen, United States), following the manufacturer’s protocol. For mRNA sequencing, samples were submitted to Gene Denovo Biotechnology Co. (Guangzhou, China), where RNA quality evaluation, mRNA enrichment, and cDNA library preparation were performed. The cDNA libraries were sequenced on the Illumina sequencing platform Novaseq6000 by Gene Denovo Biotechnology Co., Ltd. (Guangzhou, China). RNA differential expression was analyzed using DESeq2 [7] software. Transcripts with a false discovery rate (FDR) < 0.05 and absolute fold change (FC) ≥ 2 were considered s differentially expressed genes/transcripts. The upregulation or downregulation of genes depended on the change in mean value of Fragments Per Kilobase of transcript per Million mapped reads (FPKM) in HGF-UCMSCs in comparison with UCMSCs. A volcanic map was drawn to display the differentially significant genes.

### Statistical analysis

Statistical analysis was performed using GraphPad Prism 8.0 (GraphPad Software, Inc. United States). Two groups were compared using an unpaired *t* test. Comparisons among more than two groups were performed using a one-way analysis of variance, followed by Tukey’s multiple comparison test. All values were expressed as the mean ± standard deviation. *P* < 0.05 was considered statistically significant.

## Results

### Cell identification and quality control

**FIGURE 2:**
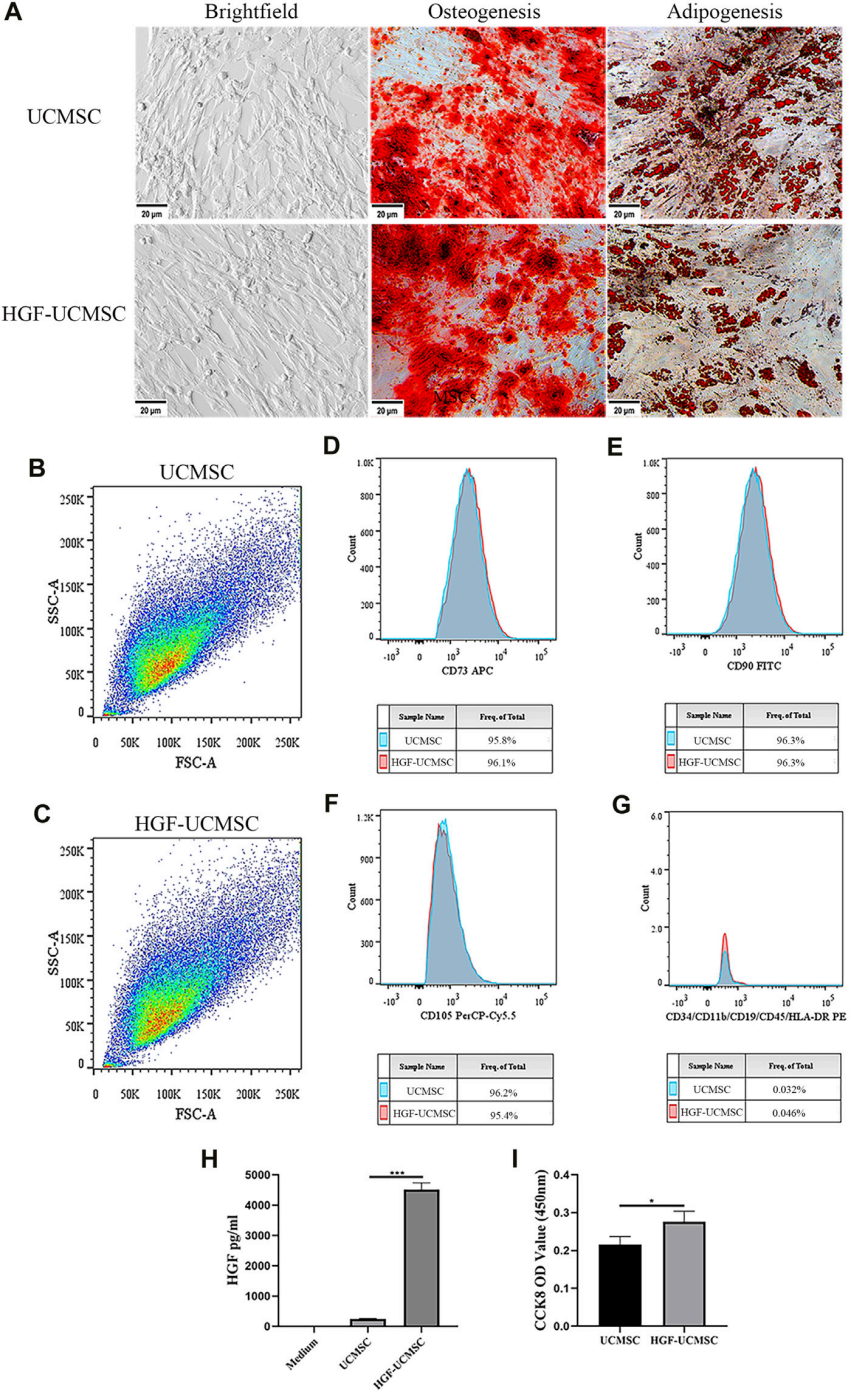
Characteristics of UCMSCs and HGF-UCMSCs. **(A)** Regularly cultured MSCs were induced into osteoblasts and adipoblasts stained with alizarin red and Oil Red O, respectively (magnification: ×100). **(B–G)** Flow cytometry was utilized to analyze the physical features and cell surface markers of MSCs **(H)** The concentration of HGF in the conditioned medium was detected using a commercial ELISA kit **(I)** The proliferation of MSCs was assessed by CCK8 assay. Values are presented as mean ± standard deviation. * *p* < 0.05; ** *p* < 0.01; *** *p* < 0.001.

In bright-field microscopy, both UCMSCs and HGF-UCMSCs were plastic-adherent and appeared spindle-shaped (Figure 2A). Ad-HGF transfection hardly changed the morphology of UCMSCs. Cultured in a specific differentiation induction medium, UCMSCs and HGF-UCMSCs differentiated into osteoblasts and adipoblasts (Figure 2A) with no obvious difference between the two groups. To identify MSC biomarkers, we performed flow cytometry. Overexpression of HGF did not alter the size and granularity of UCMSCs (Figures 2B, C). CD73, CD90, and CD105 were all positively expressed and CD34, CD11b, CD19, CD45, and HLA-DR were seldom expressed on the surface of the cells (Figures 2D–2G). Similar to the results of cell differentiation, gene modification did not influence the proportion of the markers mentioned above. To verify whether Ad-HGF transfection was successful, we measured the concentration of HGF in CM. The level of HGF was much higher in the CM of HGF-UCMSCs than that of UCMSCs (*p* < 0.0001) (Figure 2H). Moreover, HGF-UCMSCs proliferated faster than did UCMSCs (*p* = 0.0378) (Figure 2I). Collectively, the transfection of Ad-HGF promoted HGF secretion and cell proliferation without changing the basic characteristics of UCMSCs.

### Efficacy of HGF-UCMSCs in lung function

**FIGURE 3:**
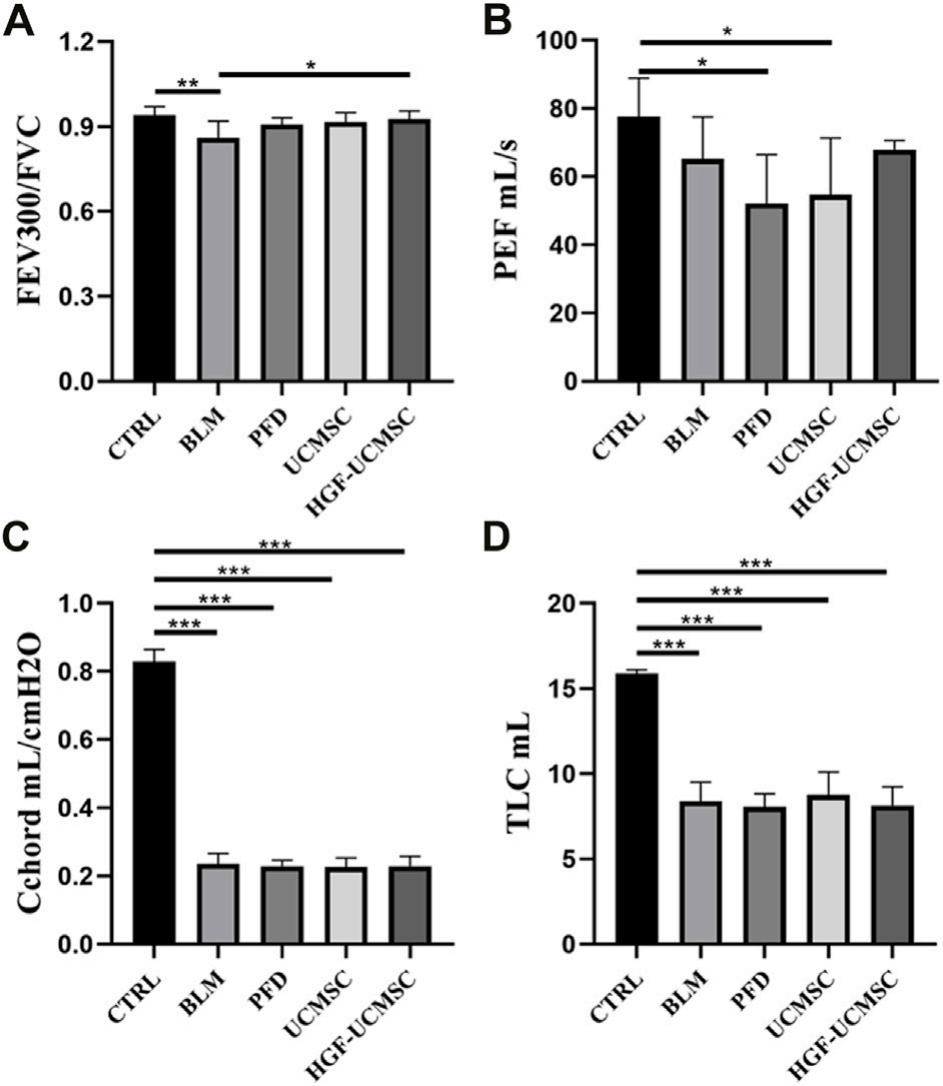
Lung function test on day 21 before euthanasia. **(A,B)** Pulmonary ventilatory function: forced expiratory volume in the first 300 m/forced vital capacity (FEV300/FVC); peak expiratory flow (PEF). **(C)** Pulmonary compliance: chord compliance (Cchord). **(D)** Pulmonary volume: total lung capacity (TLC). Values are presented in mean ± standard deviation. * *p* < 0.05; ** *p* < 0.01; *** *p* < 0.001.

Before euthanizing the rats, we evaluated the physiological function of the lungs. Compared with the CTRL group, FEV300/FVC, PEF, Cchord and TLC in the BLM group decreased significantly (*p* < 0.05) (Figure 3). The PFD, UCMSC and HGF-UCMSC groups alleviated the pulmonary function injury, but the statistical difference was only found between the BLM and HGF-UCMSC group (*p* < 0.05). The therapeutic effect was not present for PEF, Cchord and TLC in the PFD, UCMSC and HGF-UCMSC group (*p* > 0.05).

### Effect of HGF-UCMSCs on the improvement of lung structure

**FIGURE 4:**
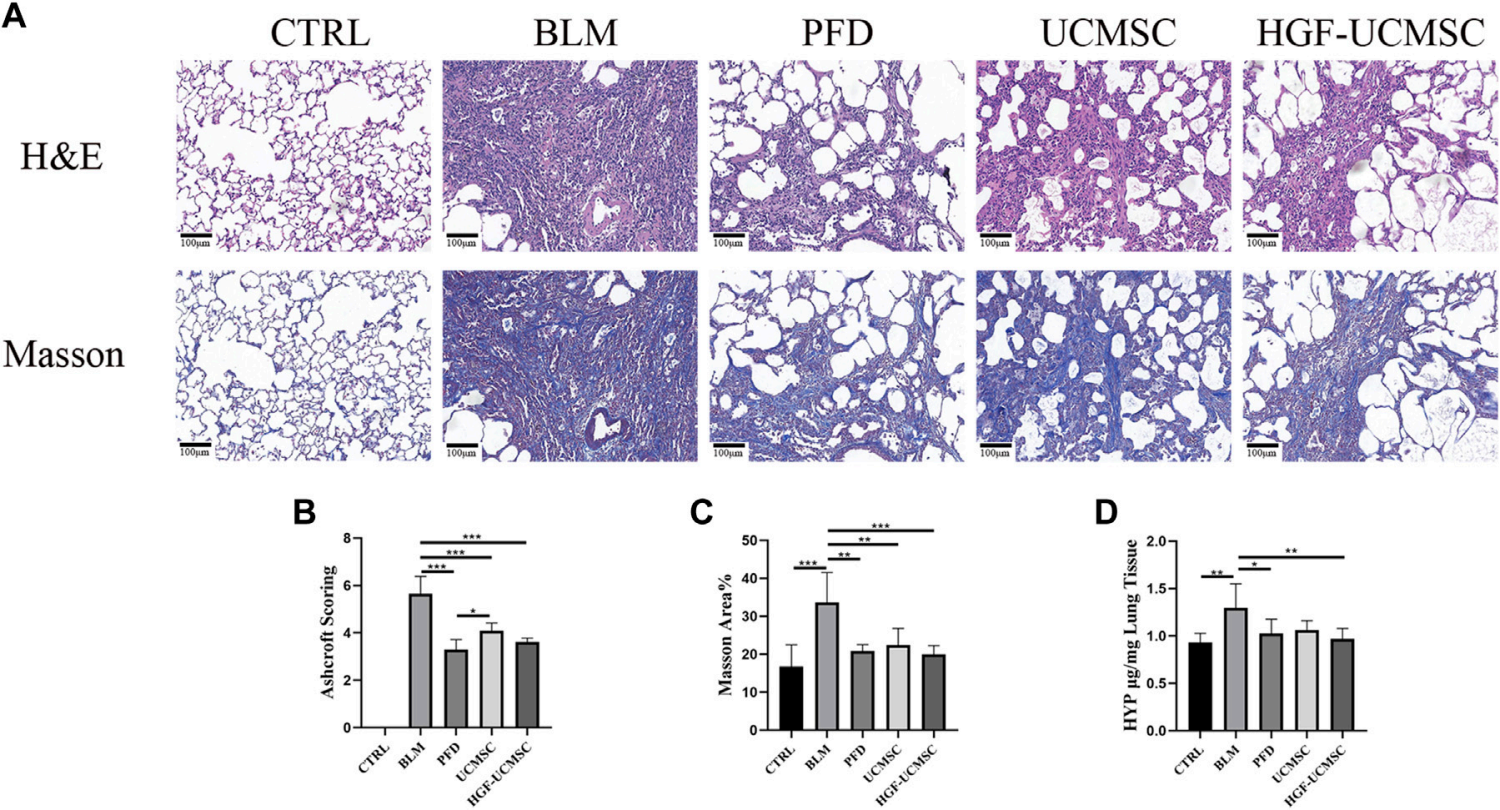
Evaluation of pulmonary histopathology. **(A)** Lung sections were stained with H&E or Masson’s trichrome (magnification: ×100). **(B)** Ashcroft scoring was performed in H&E-stained sections **(C)** The percentage positive area was calculated in Masson’s trichrome-stained sections **(D)** HYP concentrations were detected in the lungs. Values are presented in mean ± standard deviation. * *p* < 0.05; ** *p* < 0.01; *** *p* < 0.001.

The histopathology was analyzed to estimate the improvement effect on lung structure. In H&E-stained sections, pulmonary fibrosis of differing degrees was induced in the other groups but was not seen in the CTRL group. The alveolar septum was filled with mesenchymal tissue stained with “acidophilic eosin” (Figure 4A). Ashcroft scores in the BLM group were significantly higher than that in the CTRL group (*p* < 0.05) (Figure 4B). The scores were decreased in all therapeutic groups, in which the PFD group got the lowest scores followed by the HGF-UCMSC group (*p* < 0.05). In sections stained with Masson trichrome, there was a large amount of collagen abnormally present in the interstitial lung tissue (Figure 4A). Similar to the result of Ashcroft scoring, the CTRL group showed significantly less Masson area% than the BLM group (*p* < 0.05) (Figure 4C). In comparison to the BLM group, the PFD, UCMSC and HGF-UCMSC groups significantly decreased the Masson area% (*p* < 0.05). Of note, the HGF-UCMSC group had the lowest *p*-value among the treatment groups in comparison with the BLM group (*p* = 0.0005). For the evaluation of collagen content, we detected HYP levels in the lungs. A higher level of HYP was present in the BLM group than in the CTRL group (*p* < 0.05) (Figure 4D). The treatment groups had various degrees of inhibitory effect on collagen deposition. HYP was significantly decreased in PFD and HGF-MSC group than the BLM group (PFD vs. BLM, *p* = 0.0378; HGF-UCMSC vs BLM, *p* = 0.0088); the UCMSC group exhibited less HYP, with no statistical significance (*p* > 0.05). Though the statistical difference was not present between the UCMSC and HGF-UCMSC groups, it is likely that HGF modification probably promoted the anti-fibrotic effect of UCMSCs in bleomycin-induced pulmonary fibrosis.

### Influence of HGF-UCMSC treatment on lung cytokines

**FIGURE 5:**
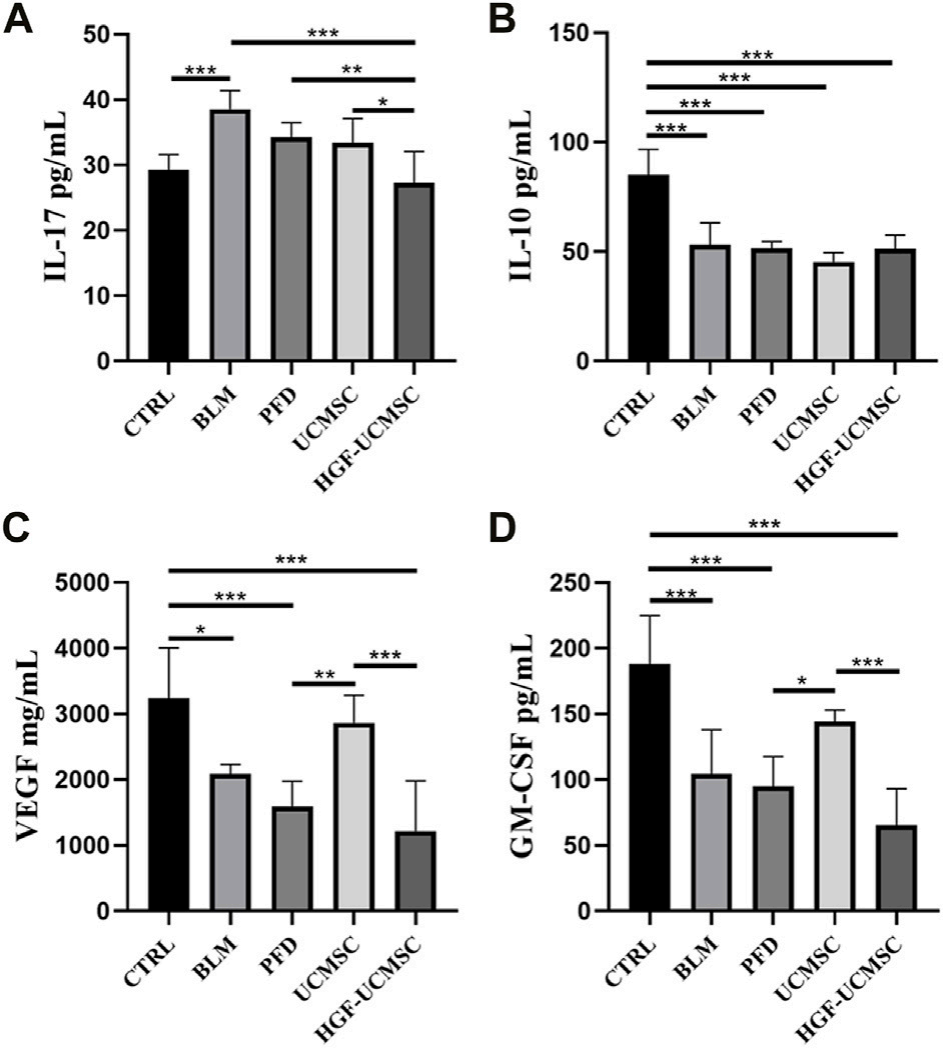
Detection of cytokines in the lungs. **(A)** Interleukin-17 (IL-17). **(B)** IL-10 **(C)** Vascular endothelial growth factor (VEGF). **(D)** Granulocyte-macrophage colony-stimulating factor (GM-CSF). Values are presented as mean ± standard deviation. * *p* < 0.05; ** *p* < 0.01; *** *p* < 0.001.

To explain why HGF-UCMSCs had better efficacy than UCMSCs in treating pulmonary fibrosis, cytokines that may be involved were detected according to previous studies (François et al., 2015). The concentration of IL-17 in the BLM group was higher than that in the CTRL group (*p* < 0.05) (Figure 5A). HGF-UCMSCs had significantly decreased levels of IL-17 compared with the BLM, PFD, and UCMSC groups (*p* < 0.05). There was no significance in the PFD and UCMSC group in comparison with the BLM group (*p* > 0.05), although these two groups showed lower levels of IL-17. Compared with the CTRL group, all groups administered with bleomycin exhibited much lower levels of IL-10, and no significance was seen among the bleomycin groups (*p* > 0.05) (Figure 5B). Significantly decreased VEGF and GM-CSF levels were observed in the BLM, PFD, and HGF-UCMSC groups, as compared with the CTRL group (*p* < 0.05) (Figures 5C, D). The UCMSC group had a numerically higher level of VEGF and GM-CSF than did the PFD and HGF-UCMSC groups (*p* < 0.05), although there was no significant difference between the UCMSC and BLM groups (*p* > 0.05). In short, IL-17 may play a role in treatment with respect to HGF-UCMSCs.

### Transcriptome difference between UCMSCs and HGF-UCMSCs

**FIGURE 6:**
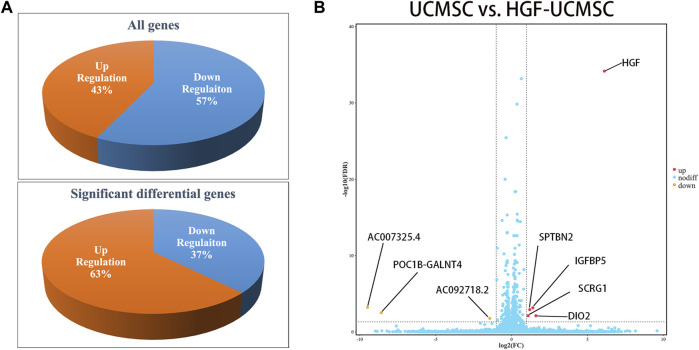
Analysis of transcriptional expression of UCMSCs and HGF- UCMSCs. **(A)** The percentage of up-regulated and down-regulated genes was calculated among all genes (upper panel) or the significant differential genes (lower panel) **(B)** Significant differential gene symbols are shown in the volcanic map.

Owing to few studies that have depicted the influence of HGF modification on UCMSCs, we conducted mRNA sequencing to uncover the transcriptional change of transfecting Ad-HGF into UCMSCs, thereby ascertaining whether HGF alone participates in treating pulmonary fibrosis. In total, 57% of genes were down-regulated and 43% of genes were up-regulated in all detected genes, without considering the statistical significance (Figure 6A). Among them, three genes (AC092718.2, POC1B-GALNT4 and AC007325.4) were significantly down-regulated, and five genes (HGF, DIO2, IGFBP5, SPTBN2 and SCRG1) were significantly up-regulated, with *p* values <0.05. (Figure 6B). As shown in Table 1, the log2(FC) value was higher and the FDR value was lower in the HGF gene than in the other genes, which suggested that the anti-fibrotic effect may be primarily owing to HGF.

**TABLE 1:** Statistics for significant differential genes.

Trend	Ensembl ID	Symbol	UCMSC mean FPKM	HGF-UCMSC mean FPKM	log2(FC)	FDR	GO cellular component	GO molecular function	GO biological process
Up regulation	ENSG00000019991	HGF	4.12	288.36	6.1286	0	extracellular region//extracellular space//membrane//. . .	serine-type endopeptidase activity//protein binding//growth factor activity//. . .	MAPK cascade//activation of MAPK activity//mitotic cell cycle//. . .
	ENSG00000211448	DIO2	0.42	1.28	1.6159	0.007803	plasma membrane//membrane//integral component of membrane	thyroxine 5'-deiodinase activity//selenium binding//oxidoreductase activity//…	selenocysteine incorporation//thyroid hormone generation//...
	ENSG00000115461	IGFBP5	0.21	0.56	1.415323	0.000722	extracellular region//extracellular space//endoplasmic reticulum lumen//…	fibronectin binding//protein binding//insulin-like growth factor binding//…	regulation of cell growth// osteoblast differentiation//signal transduction//…
	ENSG00000173898	SPTBN2	0.42	0.98	1.210778	0.00125	extracellular space//cytoplasm//cytosol//…	actin binding//structural constituent of cytoskeleton//protein binding//…	MAPK cascade//ER to Golgi vesicle-mediated transport//cytoskeleton organization//…
	ENSG00000164106	SCRG1	0.46	0.99	1.103436	0.007807	extracellular region//extracellular space//cytoplasm//…	protein binding	nervous system development//mesenchymal stem cell proliferation
Down regulation	ENSG00000260643	AC092718.2	2.09	0.78	-1.423191	0.017627	mitochondrion//glycine cleavage complex//membrane//…		glycine decarboxylation via glycine cleavage system
	ENSG00000259075	POC1B-GALNT4	0.38	0.001	-8.571121	0.003186	Golgi membrane//Golgi apparatus//membrane//…	polypeptide N-acetylgalactosami nyltransferase activity//transferase activity//transferase activity, transferring glycosyl groups//…	protein glycosylation//protein phosphopantet heinylation
	ENSG00000278817	AC007325.4	0.71	0.001	-9.473706	0.000635			

Abbreviations: HGF, hepatocyte growth factor; DIO2, iodothyronine deiodinase 2; IGFBP5, insulin like growth factor binding protein 5; SPTBN2, spectrin beta, non-erythrocytic 2; SCRG1, stimulator of chondrogenesis 1; POC1B-GALNT4, POC1B-GALNT4 readthrough.

## Discussion

Gene modification is a promising strategy in the application of MSCs in treating pulmonary fibrosis (Min et al., 2015; Lan et al., 2017). As such, individually designed MSCs may function precisely in the treatment of pulmonary fibrosis. It has been demonstrated that HGF functions as a protective protein in pulmonary fibrosis through binding its receptor, c-met, which is expressed by many types of cells (e.g., epithelial cells, fibroblasts) (Dohi et al., 2000; Watanabe et al., 2005; Gazdhar et al., 2007; Gazdhar et al., 2013; Gazdhar et al., 2018). We therefore performed HGF modification in UCMSCs in order to enhance the anti-fibrotic ability of the cells. Our data showed that the modification improved the anti-fibrotic efficacy of UCMSCs in pulmonary ventilatory function and collagen deposition in the lung.

To explore the possible mechanism of HGF-UCMSCs in treating pulmonary fibrosis, we detected the cytokines that may participate in the process. The results showed that IL-17 may be affected by the treatment of HGF-UCMSCs. Produced by T helper 17 (Th17) cells, IL-17 acts as a driver of pulmonary fibrosis (François et al., 2015; Ting et al., 2017). The deletion of IL-17 in mice ameliorated the severity of pulmonary fibrosis induced by bleomycin (Wilson et al., 2010). In our study, compared with the BLM group, all treatment groups had numerically decreased IL-17 levels but only the HGF-UCMSC group showed a significant inhibitory effect (*p* < 0.0001, Figure 6A). Thus, HGF-UCMSCs may ameliorate pulmonary fibrosis by inhibiting IL-17 in the lung. Our data also showed that significantly lower IL-17 levels were seen in the HGF-UCMSC group than those in the UCMSC group (*p* = 0.0285), which means that the inhibitory effect may be associated with HGF modification. To explore whether other anti-fibrotic factors influenced the inhibition of IL-17 apart from HGF, we performed mRNA sequencing in UCMSCs and HGF-UCMSCs. The data showed that the transcriptional level of HGF was relatively higher than that of other significantly different genes, which suggested that HGF may play the main role in inhibiting IL-17. Interestingly, HGF secreted by MSCs is reported to mediate the differentiation from CD4^+^ cells to regulatory T cells but not Th17 cells, the IL-17-producing cells (Chen et al., 2020). Therefore, we propose the possible therapeutic mechanism that HGF-UCMSCs may directly or indirectly interact with CD4^+^ cells or Th17 cells through HGF in fibrotic lungs.

Apart from HGF, the transfection of Ad-HGF also change the transcriptional expression of other genes in UCMSCs. DIO2 acts as an activator of the thyroid hormone, which is critical for the maintenance of cellular homeostasis during stress responses (Sagliocchi et al., 2019). Yu et al. (Yu et al., 2018) reported that DIO2-knockout mice exhibited more severe pulmonary fibrosis. The authors also used thyroid hormone to treat pulmonary fibrosis in mice; their results showed increased survival and resolved lung fibrosis. Insulin-like growth factor binding protein 5 (IGFBP5) was reported as a pro-fibrotic factor in pulmonary fibrosis (Yasuoka et al., 2006). The expression of the human IGFBP5 gene in transgenic mice induced the up-regulation of ECM genes in the lungs (Nguyen et al., 2021). The role of crapie responsive gene 1 (SCRG1) in pulmonary fibrosis is unknown, but it was reported that SCRG1 is associated with the stemness of MSCs (Chosa and Ishisaki, 2018). The relationship between other significantly changed genes and pulmonary fibrosis or MSCs remains unclear in the present.

Our study had some limitations. First, HGF-UCMSCs did not show significantly anti-fibrotic effect in the comparison to UCMSCs, albeit the administration of HGF-UCMSCs but not wild-type UCMSCs significantly improved FEV300/FVC and lung HYP level in our study. This might be attributed to the use of bleomycin in over dosage that is higher than the dosage reported in the published papers (Lee et al., 2010; Rathinasabapathy et al., 2016; Chu et al., 2019). Second, we did not investigate the specific mechanism of the interaction between HGF-UCMSCs and IL-17-producing cells. Thus, *in vitro* and *in vivo* experiments are needed to confirm whether IL-17-producing cells are the targets of HGF and whether IGFBP5 and SCRG1 participate in the treatment of pulmonary fibrosis. Thrid, we did not explore how HGF-UCMSCs function at different stages after treatment and whether there is an appropriate time window for HGF-UCMSC treatment for better therapeutic efficacy. These may be the keys to reverse fibrosis. Fourth, it remains unclear how the levels of VEGF and GM-CSFin lungs were decreased by HGF-UCMSCs in comparison to UCMSCs. To sample in earlier timing after the administration of HGF-UCMSCs and to determine the main cells secreting VEGF and GM-CSF might be important to reveal the possible mechanism.

## Conclusion

In this study, we confirmed that treatment with UCMSCs or HGF-UCMSCs could alleviate pulmonary fibrosis caused by bleomycin in mice. Furthermore, the enhancement of HGF secretion may improve the anti-fibrotic effect of UCMSCs. The improved anti-fibrotic effect may be associated with the inhibition of IL-17 in the lungs.

## Data Availability

The original contributions presented in the study are publicly available. This data can be found here: https://www.ncbi.nlm.nih.gov/sra/PRJNA915313, accession number PRJNA915313.

## References

[B1] Al Naem M. Bourebaba L. Kucharczyk K. Röcken M. Marycz K. (2020). Therapeutic mesenchymal stromal stem cells: Isolation, characterization and role in equine regenerative medicine and metabolic disorders. Stem Cell. Rev. Rep. 16 (2), 301–322. 10.1007/s12015-019-09932-0 31797146

[B2] Ashcroft T. Simpson J. M. Timbrell V. (1988). Simple method of estimating severity of pulmonary fibrosis on a numerical scale. J. Clin. Pathol. 41 (4), 467–470. 10.1136/jcp.41.4.467 3366935 PMC1141479

[B3] Balasubramanian S. Venugopal P. Sundarraj S. Zakaria Z. Majumdar A. S. Ta M. (2012). Comparison of chemokine and receptor gene expression between Wharton's jelly and bone marrow-derived mesenchymal stromal cells. Cytotherapy 14 (1), 26–33. 10.3109/14653249.2011.605119 22091833

[B4] Cahill E. F. Kennelly H. Carty F. Mahon B. P. English K. (2016). Hepatocyte growth factor is required for mesenchymal stromal cell protection against bleomycin-induced pulmonary fibrosis. Stem Cells Transl. Med. 5 (10), 1307–1318. 10.5966/sctm.2015-0337 27388243 PMC5031177

[B5] Cao X.-P. Han D.-M. Zhao L. Guo Z.-K. Xiao F.-J. Zhang Y.-K. (2016). Hepatocyte growth factor enhances the inflammation-alleviating effect of umbilical cord–derived mesenchymal stromal cells in a bronchiolitis obliterans model. Cytotherapy 18 (3), 402–412. 10.1016/j.jcyt.2015.12.006 26857230

[B6] Chambers D. C. Enever D. Ilic N. Sparks L. Whitelaw K. Ayres J. (2014). A phase 1b study of placenta‐derived mesenchymal stromal cells in patients with idiopathic pulmonary fibrosis. Respirology 19 (7), 1013–1018. 10.1111/resp.12343 25039426

[B7] Chen Q. H. Wu F. Liu L. Chen H. B. Yu L. N. Wang H. L. (2020). Mesenchymal stem cells regulate the Th17/Treg cell balance partly through hepatocyte growth factor *in vitro* . Stem Cell. Res. Ther. 11 (1), 91. 10.1186/s13287-020-01612-y 32111238 PMC7049226

[B8] Chen S. Cui G. Peng C. Lavin M. F. Sun X. Zhang E. (2018). Transplantation of adipose-derived mesenchymal stem cells attenuates pulmonary fibrosis of silicosis via anti-inflammatory and anti-apoptosis effects in rats. Stem Cell. Res. Ther. 9 (1), 110–112. 10.1186/s13287-018-0846-9 29673394 PMC5909257

[B9] Chen Y. Qian H. Zhu W. Zhang X. Yan Y. Ye S. (2011). Hepatocyte growth factor modification promotes the amelioration effects of human umbilical cord mesenchymal stem cells on rat acute kidney injury. Stem Cells Dev. 20 (1), 103–113. 10.1089/scd.2009.0495 20446811

[B59] Chosa N. Ishisaki A. (2018). Two novel mechanisms for maintenance of stemness in mesenchymal stem cells: SCRG1/BST1 axis and cell–cell adhesion through N-cadherin. Jpn. Dent. Sci. Rev. 54 (1), 37–44. 10.1016/j.jdsr.2017.10.001 29629000 PMC5884250

[B10] Chu K.-A. Wang S.-Y. Yeh C.-C. Fu T.-W. Fu Y.-Y. Ko T.-L. (2019). Reversal of bleomycin-induced rat pulmonary fibrosis by a xenograft of human umbilical mesenchymal stem cells from Wharton's jelly. Theranostics 9 (22), 6646–6664. 10.7150/thno.33741 31588241 PMC6771241

[B11] Dohi M. Hasegawa T. Yamamoto K. Marshall B. C. (2000). Hepatocyte growth factor attenuates collagen accumulation in a murine model of pulmonary fibrosis. Am. J. Respir. Crit. Care Med. 162 (6), 2302–2307. 10.1164/ajrccm.162.6.9908097 11112155

[B12] Dong J. Yu X. Porter D. W. Battelli L. A. Kashon M. L. Ma Q. (2016). Common and distinct mechanisms of induced pulmonary fibrosis by particulate and soluble chemical fibrogenic agents. Arch. Toxicol. 90 (2), 385–402. 10.1007/s00204-015-1589-3 26345256 PMC4749430

[B13] Dong L.-H. Jiang Y.-Y. Liu Y.-J. Cui S. Xia C.-C. Qu C. (2015). The anti-fibrotic effects of mesenchymal stem cells on irradiated lungs via stimulating endogenous secretion of HGF and PGE2. Sci. Rep. 5 (1), 8713–8810. 10.1038/srep08713 25736907 PMC4348621

[B14] Dove E. P. Olson A. L. Glassberg M. K. (2019). Trends in idiopathic pulmonary fibrosis–related mortality in the United States: 2000–2017. Am. J. Respir. Crit. Care Med. 200 (7), 929–931. 10.1164/rccm.201905-0958LE 31225965

[B15] El Omar R. Beroud J. Stoltz J.-F. Menu P. Velot E. Decot V. (2014). Umbilical cord mesenchymal stem cells: The new gold standard for mesenchymal stem cell-based therapies? Tissue Eng. Part B Rev. 20 (5), 523–544. 10.1089/ten.teb.2013.0664 24552279

[B16] François A. Gombault A. Villeret B. Alsaleh G. Fanny M. Gasse P. (2015). B cell activating factor is central to bleomycin-and IL-17-mediated experimental pulmonary fibrosis. J. Autoimmun. 56, 1–11. 10.1016/j.jaut.2014.08.003 25441030

[B17] Gazdhar A. Fachinger P. van Leer C. Pierog J. Gugger M. Friis R. (2007). Gene transfer of hepatocyte growth factor by electroporation reduces bleomycin-induced lung fibrosis. Am. J. Physiol. Lung Cell. Mol. Physiol. 292, L529–L536. 10.1152/ajplung.00082.2006 17056705

[B18] Gazdhar A. Fytianos K. Knudsen L. Schliep R. Mueller S. Geiser T. (2018). Electroporation of Hepatocyte growth factor to the lung induces migration of bone marrow mesenchymal stem cells and reduces lung fibrosis. Eur. Respir. Soc. 10.1183/13993003.congress-2018.PA593

[B19] Gazdhar A. Temuri A. Knudsen L. Gugger M. Schmid R. A. Ochs M. (2013). Targeted gene transfer of hepatocyte growth factor to alveolar type II epithelial cells reduces lung fibrosis in rats. Hum. Gene Ther. 24 (1), 105–116. 10.1089/hum.2012.098 23134111

[B20] George P. M. Patterson C. M. Reed A. K. Thillai M. (2019). Lung transplantation for idiopathic pulmonary fibrosis. Lancet. Respir. Med. 7 (3), 271–282. 10.1016/S2213-2600(18)30502-2 30738856

[B21] Glassberg M. K. Minkiewicz J. Toonkel R. L. Simonet E. S. Rubio G. A. DiFede D. (2017). Allogeneic human mesenchymal stem cells in patients with idiopathic pulmonary fibrosis via intravenous delivery (AETHER): a phase I safety clinical trial. Chest 151 (5), 971–981. 10.1016/j.chest.2016.10.061 27890713 PMC6026255

[B22] Habgood A. N. Tatler A. L. Porte J. Wahl S. M. Laurent G. J. John A. E. (2016). Secretory leukocyte protease inhibitor gene deletion alters bleomycin-induced lung injury, but not development of pulmonary fibrosis. Lab. Investig. 96 (6), 623–631. 10.1038/labinvest.2016.40 26974397 PMC4884449

[B23] Han Y.-F. Tao R. Sun T.-J. Chai J.-K. Xu G. Liu J. (2013). Optimization of human umbilical cord mesenchymal stem cell isolation and culture methods. Cytotechnology 65 (5), 819–827. 10.1007/s10616-012-9528-0 23306781 PMC3967601

[B24] Huang K. Kang X. Wang X. Wu S. Xiao J. Li Z. (2015). Conversion of bone marrow mesenchymal stem cells into type II alveolar epithelial cells reduces pulmonary fibrosis by decreasing oxidative stress in rats. Mol. Med. Rep. 11 (3), 1685–1692. 10.3892/mmr.2014.2981 25411925 PMC4270324

[B25] Hutchinson J. Fogarty A. Hubbard R. McKeever T. (2015). Global incidence and mortality of idiopathic pulmonary fibrosis: A systematic review. Eur. Respir. J. 46 (3), 795–806. 10.1183/09031936.00185114 25976683

[B26] Hutchinson J. P. McKeever T. M. Fogarty A. W. Navaratnam V. Hubbard R. B. (2014). Increasing global mortality from idiopathic pulmonary fibrosis in the twenty-first century. Ann. Am. Thorac. Soc. 11 (8), 1176–1185. 10.1513/AnnalsATS.201404-145OC 25165873

[B27] Kim J.-H. Jo C. H. Kim H.-R. Hwang Y.-i. (2018). Comparison of immunological characteristics of mesenchymal stem cells from the periodontal ligament, umbilical cord, and adipose tissue. Stem Cells Int. 2018, 8429042. 10.1155/2018/8429042 29760736 PMC5901833

[B28] King T. E. Jr Bradford W. Z. Castro-Bernardini S. Fagan E. A. Glaspole I. Glassberg M. K. (2014). A phase 3 trial of pirfenidone in patients with idiopathic pulmonary fibrosis. N. Engl. J. Med. 370 (22), 2083–2092. 10.1056/NEJMoa1402582 24836312

[B29] Kotani T. Masutani R. Suzuka T. Oda K. Makino S. Ii M. (2017). Anti-inflammatory and anti-fibrotic effects of intravenous adipose-derived stem cell transplantation in a mouse model of bleomycin-induced interstitial pneumonia. Sci. Rep. 7 (1), 14608–14610. 10.1038/s41598-017-15022-3 29097816 PMC5668313

[B30] Lan Y.-W. Theng S.-M. Huang T.-T. Choo K.-B. Chen C.-M. Kuo H.-P. (2017). Oncostatin M-preconditioned mesenchymal stem cells alleviate bleomycin-induced pulmonary fibrosis through paracrine effects of the hepatocyte growth factor. Stem Cells Transl. Med. 6 (3), 1006–1017. 10.5966/sctm.2016-0054 28297588 PMC5442768

[B31] Lederer D. J. Martinez F. J. (2018). Idiopathic pulmonary fibrosis. N. Engl. J. Med. 378 (19), 1811–1823. 10.1056/NEJMra1705751 29742380

[B32] Lee S.-H. Jang A.-S. Kim Y.-E. Cha J.-Y. Kim T.-H. Jung S. (2010). Modulation of cytokine and nitric oxide by mesenchymal stem cell transfer in lung injury/fibrosis. Respir. Res. 11 (1), 16–14. 10.1186/1465-9921-11-16 20137099 PMC2827393

[B33] Lee Y. H. Suzuki Y. J. Griffin A. J. Day R. M. (2008). Hepatocyte growth factor regulates cyclooxygenase-2 expression via β-catenin, Akt, and p42/p44 MAPK in human bronchial epithelial cells. Am. J. Physiol. Lung Cell. Mol. Physiol. 294 (4), L778–L786. 10.1152/ajplung.00410.2007 18245266 PMC2427436

[B34] Li J. Zheng C.-Q. Li Y. Yang C. Lin H. Duan H.-G. (2015). Hepatocyte growth factor gene-modified mesenchymal stem cells augment sinonasal wound healing. Stem Cells Dev. 24 (15), 1817–1830. 10.1089/scd.2014.0521 25835956 PMC4507357

[B35] Madrigal M. Rao K. S. Riordan N. H. (2014). A review of therapeutic effects of mesenchymal stem cell secretions and induction of secretory modification by different culture methods. J. Transl. Med. 12 (1), 260–314. 10.1186/s12967-014-0260-8 25304688 PMC4197270

[B36] Marmotti A. Mattia S. Castoldi F. Barbero A. Mangiavini L. Bonasia D. E. (2017). Allogeneic umbilical cord-derived mesenchymal stem cells as a potential source for cartilage and bone regeneration: An *in vitro* study. Stem Cells Int. 2017, 1732094. 10.1155/2017/1732094 29358953 PMC5735324

[B37] Min F. Gao F. Li Q. Liu Z. (2015). Therapeutic effect of human umbilical cord mesenchymal stem cells modified by angiotensin-converting enzyme 2 gene on bleomycin-induced lung fibrosis injury. Mol. Med. Rep. 11 (4), 2387–2396. 10.3892/mmr.2014.3025 25435005 PMC4337478

[B38] Mizuno S. Matsumoto K. Li M.-Y. Nakamura T. (2005). HGF reduces advancing lung fibrosis in mice: A potential role for MMP‐dependent myofibroblast apoptosis. Faseb J. 19 (6), 580–582. 10.1096/fj.04-1535fje 15665032

[B39] Nadri S. Soleimani M. Hosseni R. H. Massumi M. Atashi A. Izadpanah R. (2002). An efficient method for isolation of murine bone marrow mesenchymal stem cells. Int. J. Dev. Biol. 51 (8), 723–729. 10.1387/ijdb.072352ns 17939119

[B40] Navaratnam V. Hubbard R. B. (2019). The mortality burden of idiopathic pulmonary fibrosis in the United Kingdom. Am. J. Respir. Crit. Care Med. 200 (2), 256–258. 10.1164/rccm.201902-0467LE 30973756

[B60] Nguyen X.-X. Renaud L. Feghali-Bostwick C. (2021). Identification of Impacted Pathways and Transcriptomic Markers as Potential Mediators of Pulmonary Fibrosis in Transgenic Mice Expressing Human IGFBP5. Int. J. Moi. Sci. 22 (22), 12609. 10.3390/ijms222212609 PMC8619832 34830489

[B41] Ocansey D. K. W. Pei B. Yan Y. Qian H. Zhang X. Xu W. (2020). Improved therapeutics of modified mesenchymal stem cells: An update. J. Transl. Med. 18 (1), 42–14. 10.1186/s12967-020-02234-x 32000804 PMC6993499

[B42] Prasanna S. J. Gopalakrishnan D. Shankar S. R. Vasandan A. B. (2010). Pro-inflammatory cytokines, IFNgamma and TNFalpha, influence immune properties of human bone marrow and Wharton jelly mesenchymal stem cells differentially. PLoS One 5 (2), e9016. 10.1371/journal.pone.0009016 20126406 PMC2814860

[B43] Raghu G. Chen S.-Y. Yeh W.-S. Maroni B. Li Q. Lee Y.-C. (2014). Idiopathic pulmonary fibrosis in US medicare beneficiaries aged 65 years and older: Incidence, prevalence, and survival, 2001–11. Lancet. Respir. Med. 2 (7), 566–572. 10.1016/S2213-2600(14)70101-8 24875841

[B44] Raghu G. Rochwerg B. Zhang Y. Garcia C. A. C. Azuma A. Behr J. (2015). An official ATS/ERS/JRS/ALAT clinical practice guideline: Treatment of idiopathic pulmonary fibrosis. An update of the 2011 clinical practice guideline. Am. J. Respir. Crit. Care Med. 192 (2), e3–e19. 10.1164/rccm.201506-1063ST 26177183

[B45] Rathinasabapathy A. Bruce E. Espejo A. Horowitz A. Sudhan D. R. Nair A. (2016). Therapeutic potential of adipose stem cell‐derived conditioned medium against pulmonary hypertension and lung fibrosis. Br. J. Pharmacol. 173 (19), 2859–2879. 10.1111/bph.13562 27448286 PMC5275771

[B46] Richeldi L. Collard H. R. Jones M. G. (2017). Idiopathic pulmonary fibrosis. Lancet 389 (10082), 1941–1952. 10.1016/S0140-6736(17)30866-8 28365056

[B47] Richeldi L. Du Bois R. M. Raghu G. Azuma A. Brown K. K. Costabel U. (2014). Efficacy and safety of nintedanib in idiopathic pulmonary fibrosis. N. Engl. J. Med. 370 (22), 2071–2082. 10.1056/NEJMoa1402584 24836310

[B48] Sagliocchi S. Cicatiello A. G. Di Cicco E. Ambrosio R. Miro C. Di Girolamo D. (2019). The thyroid hormone activating enzyme, type 2 deiodinase, induces myogenic differentiation by regulating mitochondrial metabolism and reducing oxidative stress. Redox Biol. 24, 101228. 10.1016/j.redox.2019.101228 31153038 PMC6543119

[B49] Shukla M. N. Rose J. L. Ray R. Lathrop K. L. Ray A. Ray P. (2009). Hepatocyte growth factor inhibits epithelial to myofibroblast transition in lung cells via Smad7. Am. J. Respir. Cell. Mol. Biol. 40 (6), 643–653. 10.1165/rcmb.2008-0217OC 18988920 PMC2689916

[B50] Soleimani M. Nadri S. (2009). A protocol for isolation and culture of mesenchymal stem cells from mouse bone marrow. Nat. Protoc. 4 (1), 102–106. 10.1038/nprot.2008.221 19131962

[B51] Ting W. Yuan L. Jing-Feng Z. Zhen-Shun C. Aamir A. (2017). Interleukin-17 induces human alveolar epithelial to mesenchymal cell transition via the TGF-β1 mediated Smad2/3 and ERK1/2 activation. Plos One 12 (9), e0183972. 10.1371/journal.pone.0183972 28873461 PMC5584923

[B52] Tran T. Šterclová M. Mogulkoc N. Lewandowska K. Müller V. Hájková M. (2020). The European MultiPartner IPF registry (EMPIRE): Validating long-term prognostic factors in idiopathic pulmonary fibrosis. Respir. Res. 21 (1), 11–19. 10.1186/s12931-019-1271-z 31915023 PMC6951015

[B53] Tzouvelekis A. Paspaliaris V. Koliakos G. Ntolios P. Bouros E. Oikonomou A. (2013). A prospective, non-randomized, no placebo-controlled, phase Ib clinical trial to study the safety of the adipose derived stromal cells-stromal vascular fraction in idiopathic pulmonary fibrosis. J. Transl. Med. 11 (1), 1–13. 10.1186/1479-5876-11-171 23855653 PMC3722100

[B54] Wang H. Yang Y.-F. Zhao L. Xiao F.-J. Zhang Q.-W. Wen M.-L. (2013). Hepatocyte growth factor gene-modified mesenchymal stem cells reduce radiation-induced lung injury. Hum. Gene Ther. 24 (3), 343–353. 10.1089/hum.2012.177 23458413

[B55] Watanabe M. Ebina M. Orson F. M. Nakamura A. Kubota K. Koinuma D. (2005). Hepatocyte growth factor gene transfer to alveolar septa for effective suppression of lung fibrosis. Mol. Ther. 12 (1), 58–67. 10.1016/j.ymthe.2005.02.019 15963921

[B56] Wilson M. S. Madala S. K. Ramalingam T. R. Gochuico B. R. Rosas I. O. Cheever A. W. (2010). Bleomycin and IL-1beta-mediated pulmonary fibrosis is IL-17A dependent. J. Exp. Med. 207 (3), 535–552. 10.1084/jem.20092121 20176803 PMC2839145

[B61] Yasuoka H. Zhou Z. Pilewski J. M. Oury T. D. Choi A. M. K. Feghali-Bostwick C. A. (2006). Insulin-like growth factor-binding protein-5 induces pulmonary fibrosis and triggers mononuclear cellular infiltration. Am. J. Pathol. 169 (5), 1633–1642. 10.2353/ajpath.2006.060501 17071587 PMC1780193

[B57] Yin F. Wang W.-Y. Mao L.-C. Cai Q.-Q. Jiang W.-H. (2020). Effect of human umbilical cord mesenchymal stem cells transfected with HGF on TGF-β1/Smad signaling pathway in carbon tetrachloride-induced liver fibrosis rats. Stem Cells Dev. 29 (21), 1395–1406. 10.1089/scd.2020.0060 32867602

[B58] Yu G. Tzouvelekis A. Wang R. Herazo-Maya J. D. Ibarra G. H. Srivastava A. (2018). Thyroid hormone inhibits lung fibrosis in mice by improving epithelial mitochondrial function. Nat. Med. 24 (1), 39–49. 10.1038/nm.4447 29200204 PMC5760280

